# Derivation and validation of a new prediction score for bacteremia in the emergency department

**DOI:** 10.1038/s41598-026-42246-z

**Published:** 2026-03-06

**Authors:** Hirofumi Ohno, Jin Takahashi, Sayumi Kato, Kenta Ishii, Kazuhiro Hiramatsu

**Affiliations:** 1 Department of Emergency Medicine, Toyohashi Municipal Hospital, 50 Hachiken-nishi, Aotake-cho, Toyohashi, Aichi 441-8570 Japan; 2 Department of Emergency and Critical Care Medicine, Tokyo Bay Urayasu Ichikawa Medical Center, 3-4-32, Todaijima, Urayasu, Chiba 279-0001 Japan; 3 Department of Surgery, Teikyo University Hospital, 2-11-1, Kaga, Itabashi-ku, Tokyo, 173-8606 Japan

**Keywords:** Bacteremia, Prediction score, Emergency department, Biomarkers, Diseases, Medical research, Signs and symptoms, Infectious-disease diagnostics

## Abstract

Early detection and treatment of bacteremia are crucial; however, blood culture takes days. We aimed to develop a prediction score using objective emergency department (ED)-available parameters. This single-center, retrospective cohort study included patients aged ≥ 16 years admitted to our ED with suspected bacteremia. The study was divided into derivation and validation periods. Data on vital signs, routine blood tests, and blood cultures were analyzed. Using multivariate logistic regression, a prediction score was developed and validated during the derivation and validation periods, respectively. A total of 3,725 and 3,471 patients were eligible for the derivation and validation periods, respectively. Continuous variables were converted to binary forms and used in logistic regression. The resulting prediction score was: Body temperature + Platelets × 2 + Neutrophil-lymphocyte ratio × 3 + Albumin + Bilirubin × 2 + Creatinine + Lactate × 2. The area under the curve in the receiver operating characteristic curve of the prediction score in the derivation and the validation period was 0.78 (95% confidence interval: 0.76–0.80) and 0.75 (95% confidence interval: 0.73–0.78), respectively. A prediction score based on routine blood tests and vital signs can effectively predict bacteremia in ED settings.

**Supplementary Information:**

The online version contains supplementary material available at 10.1038/s41598-026-42246-z.

## Introduction

Bacteremia, the presence of viable bacteria in the bloodstream, is a serious condition associated with significant morbidity and mortality^1^. Delayed initiation of appropriate antibiotic therapy can lead to adverse outcomes, including increased mortality^2,3^. Therefore, early identification and treatment of bacteremia in patients admitted to the emergency department (ED) are important. Although the gold standard for diagnosing bacteremia is blood culture, it usually requires a few days to yield results^4,5^. Hence, it is inefficient for timely clinical decision making in the ED setting. Moreover, because blood cultures have a low positive ratio, they should be obtained from patients with a high pre-test probability of bacteremia, as unnecessary testing can be a financial burden^6^. Additionally, blood cultures exhibit the risk of yielding false-negative or false-positive results^7^. Specifically, false-positive results lead to the unnecessary use of antibiotics, additional clinical tests, increased financial costs, prolonged hospital stays, and higher mortality rates^6,8,9^.

To overcome these challenges, several studies have investigated the predictors of bacteremia and developed predictive models to identify high-risk patients early and reduce unnecessary blood cultures in low-risk patients^10–14^. These models involve vital signs, laboratory data, clinical symptoms, patient history, and comorbidities. Some of these have been well validated with good predictive performance^15,16^. However, most predictive models were developed for specific patient populations (e.g., excluding afebrile patients) and often rely on subjective parameters, such as clinical symptoms and assessments (e.g., chills and suspected endocarditis), which may vary in interpretation among clinicians^10–12^. Moreover, some models include tests that are routinely unavailable (e.g., procalcitonin), limiting their practical utility in the ED ^13,14^.

Based on previous studies, it remains unknown whether bacteremia can be predicted using only objective parameters that are commonly available in the ED. We hypothesized that a predictive score, based solely on such parameters, could effectively predict bacteremia serving as a valuable tool for early risk stratification of ED patients with suspected infection. Therefore, this study aimed to develop and validate a novel bacteremia prediction score using vital signs and routine blood tests readily available in the ED.

## Methods

### Study design and setting

This single-center, retrospective cohort study was conducted at the ED of Toyohashi Municipal Hospital. The institution is an approximately 800-bed rural community hospital with a tertiary emergency medical center covering a medical area of approximately 670 square kilometers and a population of approximately 700,000. The hospital has approximately 8,000 ambulance arrivals and 21,000 patient visits annually. The study was conducted in accordance with the Declaration of Helsinki and followed the transparent reporting of a multivariable prediction model for individual prognosis or diagnosis reporting statement for prognostic studies and did not involve the procurement of organs or tissues from prisoners. The study protocol was approved by the institutional review board of Toyohashi Municipal Hospital (approval date: May 3, 2021; approval number: 589). The requirement for informed consent was waived because of the retrospective nature of the study, which used anonymous data collected from medical records; this waiver was formally granted by the institutional review board of Toyohashi Municipal Hospital.

### Study samples and data collection

We included patients aged ≥ 16 years who visited the ED between January 2019 and March 2021, with suspected bacteremia by the attending emergency physician, and had two or more blood cultures collected. Suspicion of bacteremia was based on the clinical assessment of the attending emergency physician without any specific criteria for patient selection. Patients with missing data were excluded. The study was divided into two phases: derivation (January 2019 to March 2020) and validation (April 2020 to March 2021) periods. During both periods, vital signs were measured, and routine blood tests were performed along with blood cultures upon ED admission. Blood cultures were obtained in a uniform manner following general recommendations^17^. Two different venipuncture sites were selected, cleaned with alcohol and chlorhexidine swabs, and punctured using an aseptic technique, with at least 20 mL of blood collected from each site. Blood samples were dispensed into aerobic and anaerobic bottles and analyzed using the BacT/ALERT^®^ 3D system (bioMérieux, Marcy l’Etoile, France). Data on age, sex, medical history, vital signs on arrival, routine blood test results, blood culture results, causative organisms, source of infection, and outcomes were retrospectively collected from medical records. Vital signs included body temperature (BT) and heart rate (HR), and routine blood tests included white blood cell count (WBC), platelet count (Plt), neutrophil-lymphocyte ratio (NLR; calculated by dividing the number of neutrophils by the number of lymphocytes), albumin (Alb), bilirubin (Bil), creatinine (Cre), lactate dehydrogenase (LDH), lactate (Lac), and C-reactive protein (CRP) levels were collected as predictors. All predictors were chosen from variables repeatedly identified in previous bacteremia studies and whose results are available almost immediately in our emergency department^10–14^. Sources of infection were classified based on the clinical course in the medical records into the following categories: lung and pleura, urinary tract, biliary tract, gastrointestinal tract and peritoneum, skin and soft tissue, central nervous system, intrapelvic, head, eye, ear, nose, and trachea, bloodstream, and others including viral and fungal infections without bloodstream infection, source of unknown, and no infection. In cases with multiple sources of infection, an overlap was recorded. Conditions without infection included heart failure, acute exacerbation of chronic obstructive pulmonary disease or interstitial pneumonia, acute pancreatitis, electrolyte disturbance, stroke, hyperglycemic crisis, heat stroke, and hypothermia.

### Exposures and outcomes

Exposures were prediction scores composed of variables from vital signs and routine blood test results. The primary outcome was bacteremia, defined as a condition with a positive blood culture in at least one bottle and requiring antibiotics targeting the causative organism during the clinical course. Patients with positive blood cultures that did not require antibiotics were defined as patients with contamination. Bacteria that rarely cause bacteremia, such as coagulase-negative Staphylococci, Corynebacterium, and Bacillus species, are generally considered as contaminants, except when there is a high clinical suspicion of bacteremia^18,19^. Bacteremia or contamination was determined retrospectively based on blood culture results and the clinical course reflected in the medical records. Contamination was classified as no bacteremia.

### Statistical analysis

Patient characteristics were analyzed using the Mann-Whitney U test for continuous variables, which were described as medians with interquartile ranges (IQR), and the chi-square test for categorical variables, which were presented as percentages. Thresholds were set for each variable, and continuous parameters were converted into binary variables: BT ≥ 38.0 °C, HR ≥ 120/min, WBC ≥ 15,000/µL, Plt ≤ 100,000/µL, NLR ≥ 10, Alb ≤ 35 g/L, Bil ≥ 1.2 mg/dL, Cre ≥ 1.2 mg/dL, LDH ≥ 400 IU/L, Lac ≥ 18 mg/dL, and CRP ≥ 10 mg/dL. The thresholds were set to coincide with those used in previous studies and criteria, such as the sequential organ failure assessment (SOFA) score and the systemic inflammatory response syndrome criteria^11–13,20–22^. During the derivation period, multivariate logistic regression analysis was conducted using these binary parameters as explanatory variables and bacteremia as the dependent variable. A bacteremia prediction score was developed based on the explanatory coefficients obtained from the analysis. Each variable in the score was weighted using an integer coefficient adjusted according to its respective explanatory coefficient. The base model was constructed by identifying the smallest explanatory coefficient among the relevant variables, dividing the other coefficients by this value, rounding to the nearest integer, and assigning weights to the variables accordingly. We created several models with different numbers of variables using the backward variable-selection method, as in previous studies^23,24^, starting from the full model and repeatedly removing the least contributory variable while refitting the model at each step until the area under the receiver operating characteristic (ROC) curve (AUC) no longer differed significantly from that of the full model, as determined by the DeLong test. Internal validity was evaluated using the bootstrap method with 200 re-samples. The same analyses were performed using data from the validation period to assess the external validity. The sample size for the derivation cohort was estimated based on the rule-of-thumb of requiring 30 outcome events per candidate predictor variable to ensure sufficient statistical power and model stability in logistic regression analysis^25^. Given that we planned to include 11 variables in the model and assumed an event prevalence of 10%, the required number of events was 330, leading to a total sample size of 3,300. For the validation cohort, we ensured that the number of outcome events exceeded 200 to allow for reliable model validation^26^. The discriminative ability of the prediction score was assessed by AUC using data from the derivation period. Calibration was performed using calibration curves and slopes. A complete case analysis was adopted because missing data were assumed to be completely random, as they commonly occurred during the data collection phase and were not sufficient to influence the main results (the proportion of cases with missing data was below 1.3%, as shown in **Table ****S1**). Additionally, as a sensitivity analysis, we performed same analysis for the model with age (binary variable; ≤65 or > 65 years old) and comorbidities (binary variable; comorbidity was scored as 1 if any of the following were present: liver cirrhosis, chronic renal disease, diabetes mellitus, autoimmune disease, solid tumor, and hematologic cancer, and 0 if none were present) included as additional predictors. Statistical significance was defined as a p-value < 0.05, corresponding to the alpha level. Each test was conducted using two-tailed analyses. All analyses were performed using the R software version 4.3.2 (R Foundation for Statistical Computing, Vienna, Austria).

## Results

### Patient characteristics

**Fig. 1:**
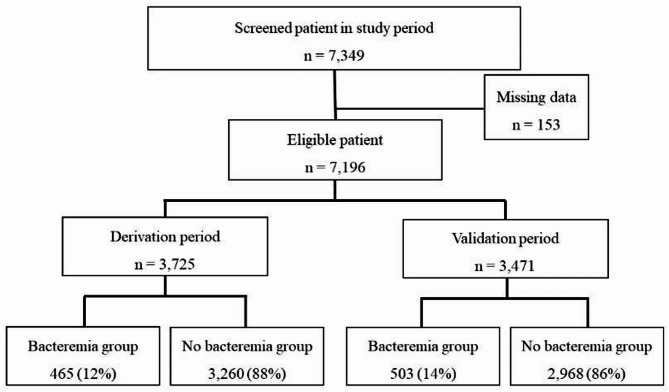
Study flow chart. During the study period, 7,349 patients with suspected bacteremia were admitted to the emergency department, and two or more blood cultures were performed. Of these, 3,725 patients in the derivation period and 3,471 patients in the validation period were included in the analysis. Among the included patients, 465 (12%) in the derivation period and 503 (14%) in the validation period were diagnosed with bacteremia.

During the study period, 7,349 patients were eligible. After excluding 153 patients with missing data, 7,196 patients were included in the final analysis (Fig. 1). Among these, 3,725 and 3,471patients were included in the derivation and validation periods, respectively. Furthermore, 465 (12%) and 503 (14%) patients were diagnosed with bacteremia, during the derivation and validation periods, respectively.

Patient characteristics and final diagnoses are presented in Table 1. Overall, the median age was 75 years (IQR, 61–84 years), and 55% were male. Patients with bacteremia had a significantly higher prevalence of diabetes mellitus and solid tumors, and a significantly higher risk of hospitalization and mortality than those without bacteremia in the derivation and validation sets (*p* < 0.001). Chronic lung disease was more common in the no bacteremia group in both sets (*p* = 0.05 and 0.04, respectively). Urinary tract infections, biliary tract infections, skin and soft tissue infections, and bloodstream infections were more frequently observed in the bacteremia group, whereas lung and pleural infections, infections of the head, eye, ear, nose, and trachea, and non-infectious conditions were more prevalent in the no bacteremia group in both sets (*p* < 0.001). The causative pathogens are listed in **Table S2**. Among gram-positive cocci, *Streptococci* and *Staphylococcus aureus* were prevalent, whereas Enterobacteriaceae, such as *Escherichia coli* or *Klebsiella* spp. were common among gram-negative rods. The contamination rates of the derivation and validation sets were 1% and 2%, respectively.

**Table 1:** Characteristics of included patients.

Derivation set (*n* = 3,725)				
	Overall	Bacteremia group *n* = 465 (12%)	No bacteremia group *n* = 3260 (88%)	*p* value
Variables				
Basic characteristics				
Age (years)	75 (61, 84)	77 (69, 85)	75 (59, 83)	< 0.01
Male sex	2050 (55)	247 (53)	1803 (55)	0.40
Comorbidities				
Heart failure	184 (5)	26 (6)	158 (5)	0.49
Chronic lung disease	287 (7)	25 (5)	262 (8)	0.05
Liver cirrhosis	55 (2)	11 (2)	44 (1)	0.10
Chronic renal disease	227 (6)	17 (4)	166 (5)	0.21
Diabetes mellitus	648 (17)	120 (26)	528 (16)	< 0.01
Autoimmune disease	186 (5)	21 (5)	165 (5)	0.73
Solid tumor	850 (23)	130 (28)	720 (22)	< 0.01
Hematologic cancer	169 (5)	16 (3)	153 (5)	0.28
Outcomes				
Hospitalization	2852 (77)	455 (98)	2397 (74)	< 0.01
Dead	273 (7)	63 (14)	210 (6)	< 0.01
Vital signs				
Body temperature (°C)	37.6 (36.9, 38.4)	38.0 (37.0, 38.9)	37.6 (36.9, 38.4)	< 0.01
Heart rate (/min)	96 (82, 110)	104 (88, 118)	95 (82, 109)	< 0.01
Laboratory data				
White blood cell count (/µL)	9,720 (6,920, 13,330)	11,450 (7,280, 16,550)	9,550 (6,888, 13,010)	< 0.01
Platelet count (/µL)	212,000 (158,000, 269,000)	178,000 (123,000, 229,000)	215,000 (162,800, 274,000)	< 0.01
Neutrophil lymphocyte ratio (%)	8.65 (4.6, 15.7)	18.3 (9.8, 32.2)	7.90 (4.3, 14.2)	< 0.01
Albumin (g/L)	3.5 (3.0, 3.9)	3.2 (2.6, 3.6)	3.5 (3.0, 3.9)	< 0.01
Bilirubin (mg/dL)	0.8 (0.5, 1.2)	1.00 (0.7, 1.6)	0.70 (0.5, 1.1)	< 0.01
Creatinine (mg/dL)	0.8 (0.6, 1.2)	1.0 (0.8, 1.7)	0.8 (0.6, 1.1)	< 0.01
Lactate dehydrogenase (IU/L)	233 (192, 293)	255 (209, 347)	230 (109, 288)	< 0.01
Lactate (mg/dL)	13 (9, 19)	18 (13, 31)	12 (9, 18)	< 0.01
C-reactive protein (mg/dL)	4.9 (1.2, 11.7)	8.8 (2.6, 19.2)	4.6 (1.1, 11.0)	< 0.01
Source of infection				
Lung and pleura	1089 (29)	53 (11)	1036 (32)	< 0.01
Urinary tract	466 (13)	158 (34)	308 (9)	< 0.01
Biliary tract	257 (7)	109 (23)	148 (5)	< 0.01
Gastrointestinal tract and peritoneum	260 (7)	37 (8)	233 (7)	0.38
Skin and soft tissue	182 (5)	37 (8)	145 (4)	< 0.01
Bloodstream	62 (2)	62 (13)	10 (0.3)	< 0.01
Central nervous system	10 (0.3)	3 (1)	7 (0.2)	0.12
Intrapelvic	13 (0.3)	0 (0)	13 (0.4)	0.40
Head, eye, ear, nose, and trachea	77 (2)	3 (1)	74 (2)	0.02
Others	558 (15)	14 (3)	544 (17)	< 0.01
No-infection	782 (21)	0 (0)	782 (24)	< 0.01
Patient condition				
Sepsis	362 (10)	138 (36)	194 (6)	< 0.01
Septic shock	75 (2)	43 (9)	32 (1)	< 0.01
Validation set (*n* = 3,471)				
	Overall	Bacteremia group *n* = 503 (14%)	No bacteremia group *n* = 2968 (86%)	p value
Variables				
Basic characteristics				
Age (years)	76 (63, 84)	77 (70, 84)	76 (62, 84)	< 0.01
Male sex	2009 (58)	261 (52)	1748 (59)	< 0.01
Comorbidities				
Heart failure	229 (7)	30 (6)	199 (7)	0.63
Chronic lung disease	250 (7)	25 (5)	225 (7)	0.04
Liver cirrhosis	58 (2)	11 (2)	47 (2)	0.35
Chronic renal disease	222 (6)	39 (8)	183 (6)	0.20
Diabetes mellitus	687 (20)	135 (27)	552 (19)	< 0.01
Autoimmune disease	159 (5)	33 (6)	126 (4)	0.03
Solid tumor	851 (25)	150 (30)	701 (24)	< 0.01
Hematologic cancer	142 (4)	17 (3)	125 (4)	0.47
Outcomes				
Hospitalization	2655 (76)	477 (95)	2178 (73)	< 0.01
Dead	308 (9)	64 (13)	244 (8)	< 0.01
Vital signs				
Body temperature (°C)	37.7 (36.9, 38.4)	38.2 (37.3, 39.1)	37.6 (36.9, 38.3)	< 0.01
Heart rate (/min)	98 (85, 112)	103 (90, 119)	98 (84, 111)	< 0.01
Laboratory data				
White blood cell count (/µL)	10,100 (7,120, 13,960)	10,860 (7560, 15355)	9905 (7018, 13660)	< 0.01
Platelet count (/µL)	205,000 (151,000, 267,000)	169,000 (125,000, 223,500)	211,000 (158,000, 273,300)	< 0.01
Neutrophil lymphocyte ratio (%)	9.2 (5.0, 17.1)	16.7 (9.1, 30.9)	8.4 (4.7, 15.3)	< 0.01
Albumin (g/L)	3.4 (2.9, 3.8)	3.2 (2.7, 3.6)	3.4 (3.0, 3.9)	< 0.01
Bilirubin (mg/dL)	0.8 (0.5, 1.1)	1.0 (0.7, 1.5)	0.7 (0.5, 1.1)	< 0.01
Creatinine (mg/dL)	0.9 (0.7, 1.2)	1.2 (0.8, 1.7)	0.8 (0.6, 1.2)	< 0.01
Lactate dehydrogenase (IU/L)	232 (191, 306)	253 (208, 329)	229 (188, 300)	< 0.01
Lactate (mg/dL)	14 (10, 21)	19 (12, 30)	13 (10, 19)	< 0.01
C-reactive protein (mg/dL)	5.1 (1.4, 12.2)	8.6 (2.6, 18.6)	4.7 (1.2, 11.3)	< 0.01
Source of infection				
Lung and pleura	817 (24)	52 (10)	765 (26)	< 0.01
Urinary tract	543 (16)	194 (39)	349 (12)	< 0.01
Biliary tract	254 (7)	86 (17)	168 (6)	< 0.01
Gastrointestinal tract and peritoneum	243 (7)	30 (6)	213 (7)	0.40
Skin and soft tissue	195 (6)	42 (8)	153 (5)	< 0.01
Bloodstream	71 (2)	67 (13)	4 (0.1)	< 0.01
Central nervous system	8 (0.2)	4 (1)	4 (0.1)	0.02
Intrapelvic	26 (0.7	4 (1)	22 (1)	0.78
Head, eye, ear, nose, and trachea	59 (2)	3 (1)	56 (2)	0.04
Others	489 (14)	24 (5)	465 (16)	< 0.01
No-infection	780 (23)	0 (0)	780 (26)	< 0.01
Patient condition				
Sepsis	433 (12)	212 (42)	201 (7)	< 0.01
Septic shock	86 (3)	50 (10)	36 (1)	< 0.01

Patient characteristics were analyzed using the Mann-Whitney U test for continuous variables, which were described as medians with interquartile ranges, and the chi-square test for categorical variables, which were presented as numbers (%).

### Development of prediction score

The results of the analyses, including the odds ratios and explanatory coefficients for each variable, are presented in Table 2. Notably, LDH contributed minimally to bacteremia prediction and was excluded from further analysis. We created multiple models whose AUCs did not differ statistically from that of the full model, and we and adopted the model with the fewest variables (**Table S3**). The final prediction score was as follows: Prediction score = NLR × 3 + Plt × 2 + Bil × 2 + Lac × 2 + Cre + BT + Alb. Each variable included in the score was assigned as 0 or 1 because it was binary.

**Table 2:** Results of univariate logistic regression analysis for each variable.

Variables	OR	95% CI	Explanatory coefficient	*p* value
Vital signs				
Body temperature ≥ 38 (°C)	1.67	1.34–2.08	0.51	< 0.01
Heart rate ≥ 120 (/min)	1.41	1.08–1.86	0.35	0.01
Routine blood tests				
White blood cell count ≥ 15,000 (/µL)	1.35	1.05–1.73	0.30	0.02
Platelet count ≤ 100,000 (/µL)	2.12	1.54–2.93	0.75	< 0.01
Neutrophil lymphocyte ratio ≥ 10	2.90	2.28–3.69	1.06	< 0.01
Albumin ≤ 35 (g/L)	1.52	1.20–1.93	0.42	< 0.01
Bilirubin ≥ 1.2 (mg/dL)	2.07	1.66–2.58	0.73	< 0.01
Creatinine ≥ 1.2 (mg/dL)	1.85	1.47–2.33	0.62	< 0.01
Lactic dehydrogenase ≥ 400 (IU/L)	1.07	0.78–1.46	0.07	0.66
Lactate ≥ 18 (mg/dL)	1.95	1.56–2.44	0.67	< 0.01
C-reactive protein ≥ 10 (mg/dL)	1.85	1.47–2.33	0.32	< 0.01

Abbreviations: OR, odds ratio; 95% CI, 95% confidence interval. All variables were analyzed using univariate logistic analysis.

### Discriminative ability, calibration, internal validation, and sensitivity analysis

**Fig. 2:**
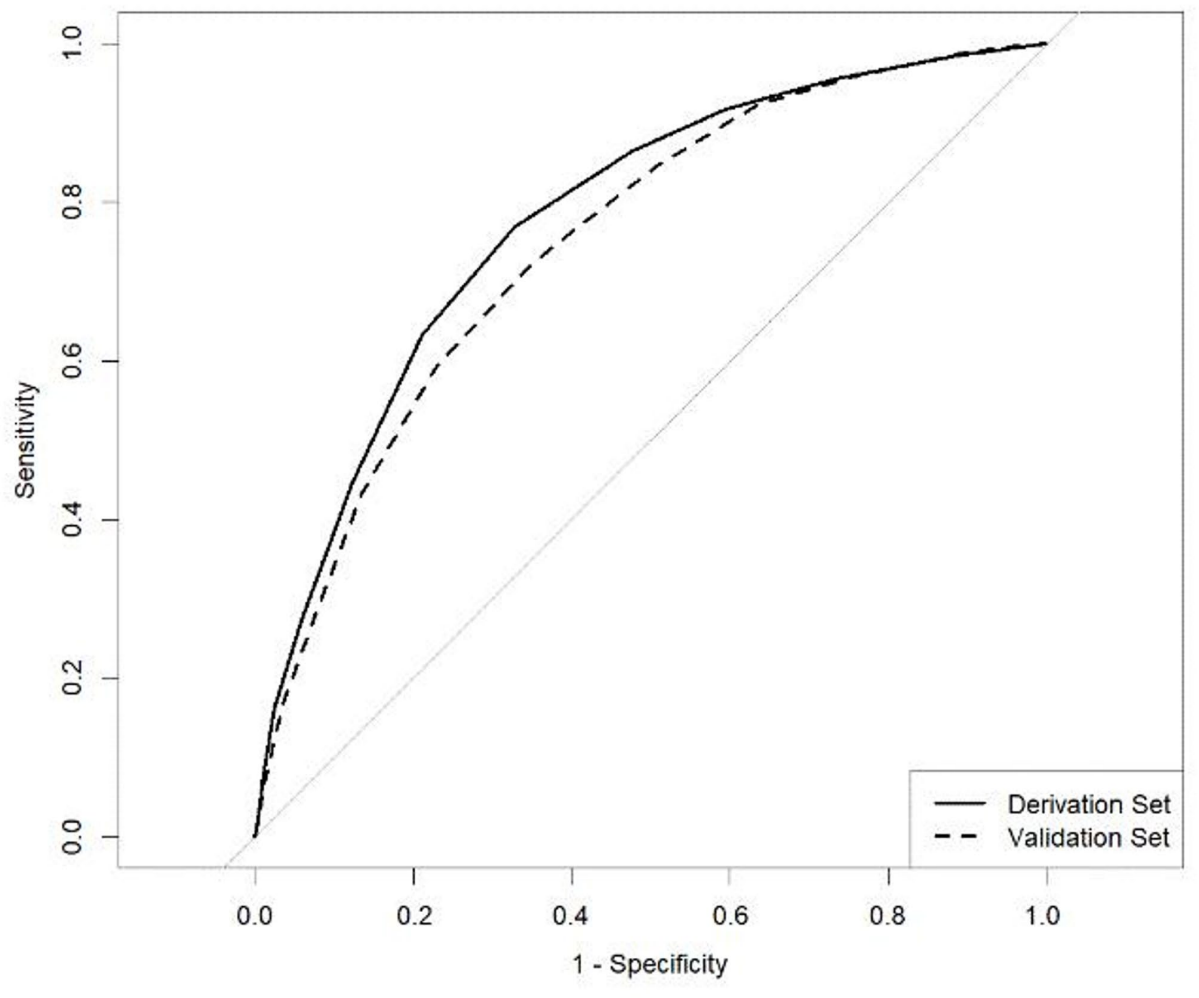
Receiver operating characteristic curve for the prediction score in the derivation and validation sets. The area under the curve of the curve is 0.78 (95% confidence interval: 0.76–0.80) and 0.75 (95% confidence interval: 0.73–0.77) in the derivation and the validation set, respectively.

The AUC of the prediction score was 0.78 (95% confidence interval [CI]: 0.76–0.80) (Fig. 2). The calibration plot for the prediction score revealed reasonable calibration with a calibration slope of 0.97 (95% CI: 0.89–1.05, **Figure ****S1**). Optimism and overfitting, calculated using the bootstrap method with 200 re-samples, were minimal, resulting in an optimism-adjusted AUC of 0.78 and a calibration slope of 1.0. The sensitivity analysis that incorporated patient age and comorbidity did not statistically change predictive performance; the AUC of the model with additional variable (0.79, 95% CI: 0.76–0.81) was not significantly different from that of the original model 1 (0.78; *p* = 0.051) (**Figure S3**).

### External validation

We performed a similar analysis using data from the validation period. The AUC of the prediction score was 0.75 (95% CI: 0.73–0.77; Fig. 2), and the calibration plot indicated adequate calibration with a calibration slope of 0.96 (95% CI: 0.87–1.05, **Figure ****S1**). Minimal optimism and overfitting were calculated using the bootstrap method with 200 re-samples, an optimism-adjusted AUC of 0.75, a calibration slope of 1.0, and calibration in the large < 0.001.

### Clinical interpretation

**Fig. 3:**
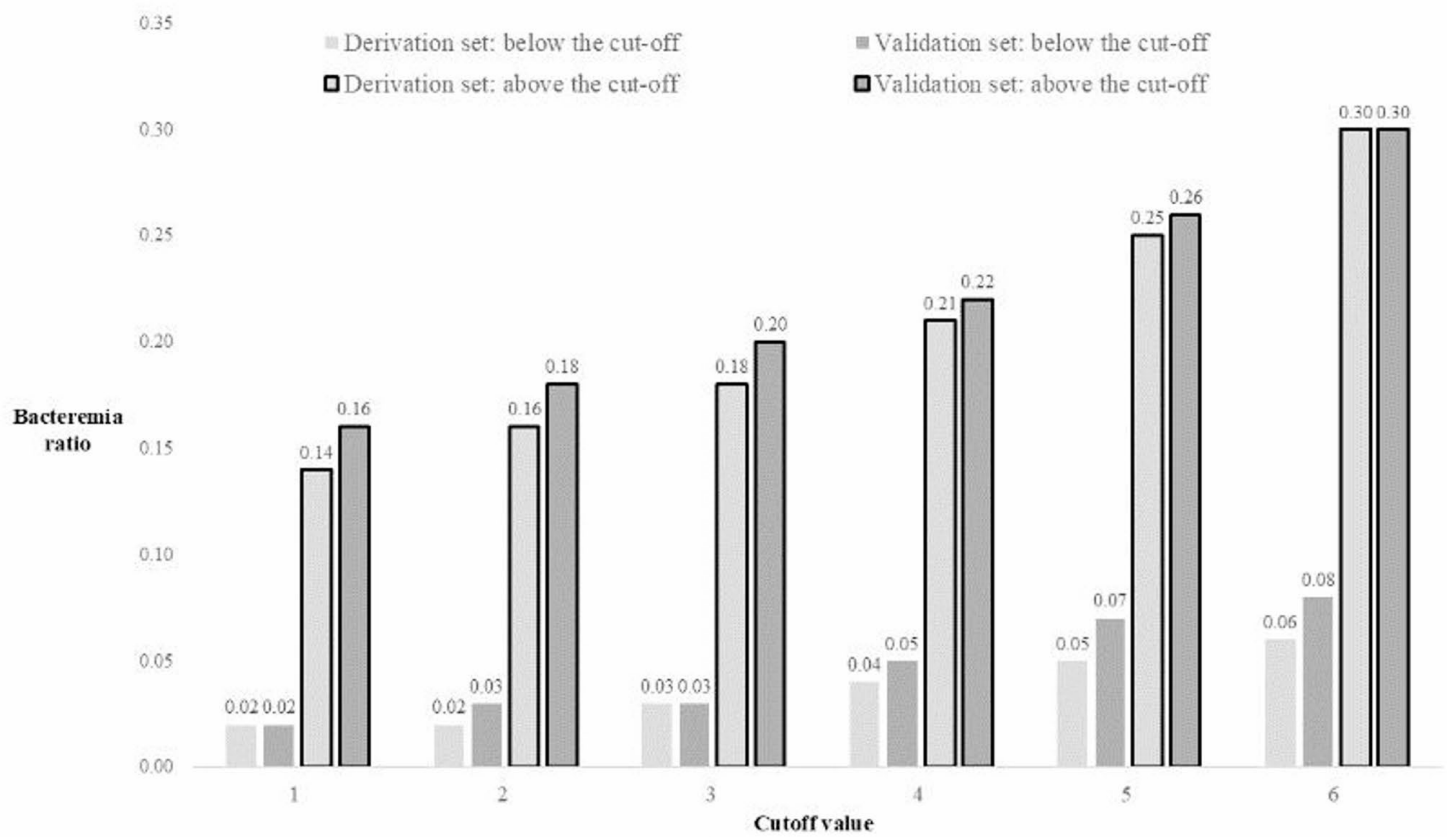
Bacteremia ratio in each cutoff value in the derivation and validation sets. The proportion of patients with bacteremia increased as the cutoff value increased, both in the group below and above the cutoff.

The sensitivity, specificity, positive predictive value (PPV), and negative predictive value (NPV) of the prediction score at different cutoff values for both the derivation and validation cohorts are presented in **Figure S2 and Table S4**. For cutoff values close to 0, the sensitivity and NPV approach approximately 1. As the cutoff increases, the specificity and PPV increase, eventually approaching 0.6 and 0.5, respectively. Figure 3 shows the bacteremia ratio for each cutoff value in both the derivation and validation sets.

## Discussions

In this study, we developed and validated a new prediction score for the early identification of bacteremia using routinely available vital signs and blood test results in an ED within a large cohort. Our findings demonstrated good discriminative ability of the prediction score in the derivation and validation sets. Furthermore, the prediction score showed reasonable calibration in both cohorts, indicating its potential for clinical application.

In clinical settings, a high prediction score indicates an elevated pretest probability of bacteremia, which may warrant prompt initiation of antibiotic therapy without waiting for blood culture results. Conversely, when the prediction score is low, the pretest probability of bacteremia is low. Depending on the patient’s condition, observation without antibiotics may be considered. We present two scenarios in a specific clinical setting.

Scenario 1: A 78-year-old man with diabetes mellitus and recent chemotherapy presented with a BT of 38.6 °C, tachycardia, and dysuria. Initial laboratory results revealed PLT: 8,000 (/µL), NLR: 20, Alb: 40 (g/L), Bil 1.1 (mg/dL), Cre 1.4 (mg/dL), and Lac 22 (mg/dL). The calculated score was 10, reflecting high pretest probability for bacteremia, as the positive likelihood ratio was 7.89. Accordingly, two sets of blood cultures were promptly obtained, antibiotic therapy was initiated, the patient was admitted to the hospital.

Scenario 2: A 46-year-old woman with no significant medical history presented with a one-day history of dysuria. She was afebrile and hemodynamically stable. Laboratory results revealed PLT 230,000 (/µL), NLR 5, Alb 40 (g/L), Bil 0.7 (mg/dL), Cre 0.8 (mg/dL), and Lac 10 (mg/dL). The calculated score was 0, reflecting low pretest probability for bacteremia, as the negative likelihood ratio was 0.13. Routine blood cultures and antibiotic therapy were deemed unnecessary unless the clinical course worsened; therefore, the patient was followed closely in the outpatient basis, without blood cultures or antibiotics.

Previous studies have developed and reported the usefulness of many prediction scores^10–14^. These prediction scores had discriminative ability with a good AUC value on the ROC curve, some of which have been well validated^14,16^. However, the items comprising these prediction scores, including patient history and clinical symptoms (e.g., chills and suspected endocarditis), are more obscure than other items and cannot be assessed in some cases, particularly in patients with impaired consciousness. The prediction score developed in this study consisted only of objective variables that were unaffected by the clinician’s assessment, making it more practical than other prediction scores previously reported in clinical settings.

Our findings may be explained by two main mechanisms. First, the prediction score included variables such as Cre, which reflects organ dysfunction due to bacteremia or comorbidities, enhancing its ability of identify at-risk patients^11,27,28^. These variables overlap with the components of SOFA score, which is used as a diagnostic criterion for sepsis^20,29^, and has demonstrated a predictive value for bacteremia, aligning with our results^22,30^. Second, the inclusion of NLR in the prediction score may explain its high accuracy, as it is useful for predicting bacteremia, even independently^21,31^. The higher explanatory coefficient of NLR underscores its importance as a key variable in the score.

The prediction score in the present study consisted only of objective variables that were instantly obtained in ED settings, allowing for evaluation without depending on the clinical assessment. This is a key strength of our study and, to the best of our knowledge, has not been proven in previous studies.

Nevertheless, this study also has several limitations. First, the criteria for blood culture collection and the definition of bacteremia are ambiguous, which may introduce selection or information bias. However, given the lack of internationally established criteria for the indication of blood culture collection or the definition of bacteremia^32^, and the reliance on clinical judgment, these factors may be considered acceptable. Second, unknown or known confounding factors were not considered. In particular, patient conditions, such as immune status, could have been considered. Additionally, the presumed source of infection is an important predictor of bacteremia; however, we could not include it in our model because we did not collect the information on the source of infection presumed before blood cultures were drawn in the emergency department. Finally, the prediction score was not validated in other settings because the study was conducted at a single center. Further research is necessary to confirm the generalizability and effectiveness of the score in various settings.

## Conclusion

The prediction score, consisting of vital signs and routine blood test results, objective and instantly obtainable in ED settings, can be useful for bacteremia prediction.

## Supplementary Information

Below is the link to the electronic supplementary material.

Supplementary Material 1

## Data Availability

The datasets generated and analyzed during the current study are not publicly available due to ethical restrictions and institutional policies that prevent the sharing of raw data to protect participant confidentiality, but are available from the corresponding author on reasonable request.
