# Supplementary material for: Derivation and validation of a new prediction score for bacteremia in the emergency department
Source: Sci Rep. 2026 Mar 6;16:12284. doi: 10.1038/s41598-026-42246-z (PMC13079805; doi:10.1038/s41598-026-42246-z)
Supplement: Supplementary file 1 — Supplementary Material 1 [file 41598_2026_42246_MOESM1_ESM.docx]

**Supplementary figures**

**Figure S1. Calibration curves for prediction scores in the derivation and validation sets.**

The prediction score was well-calibrated. The calibration slope was 0.97 (95% confidence interval: 0.89–1.05) in the derivation set, and 0.96 (95% confidence interval: 0.87–1.05) in the validation set.


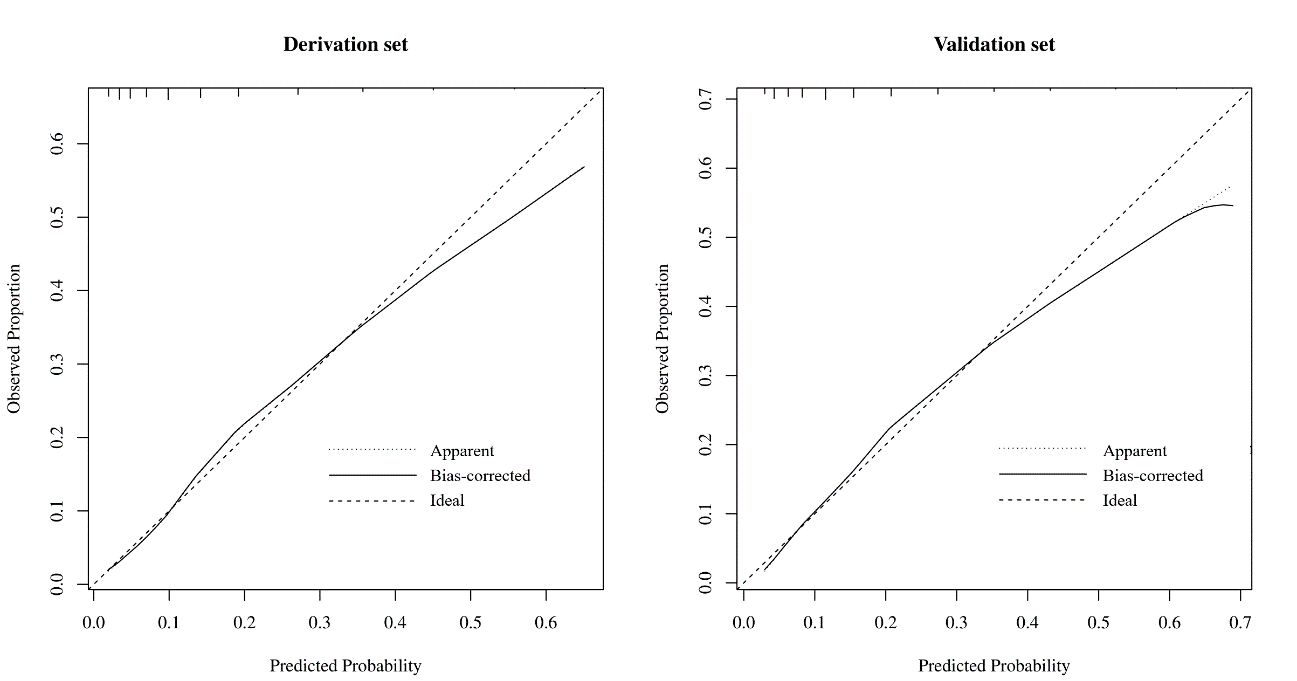


**Figure S2. Performance of the prediction score.**

The sensitivity, specificity, PPV, and NPV of each cutoff value for both the derivation and validation sets are demonstrated. For cutoff values close to 0, the sensitivity and NPV in both sets approached approximately 1. As the cutoff increases, the specificity and PPV in both sets increase, eventually approaching 0.6 and 0.5, respectively.

Abbreviations: PPV, positive predictive value; NPV, negative predictive value


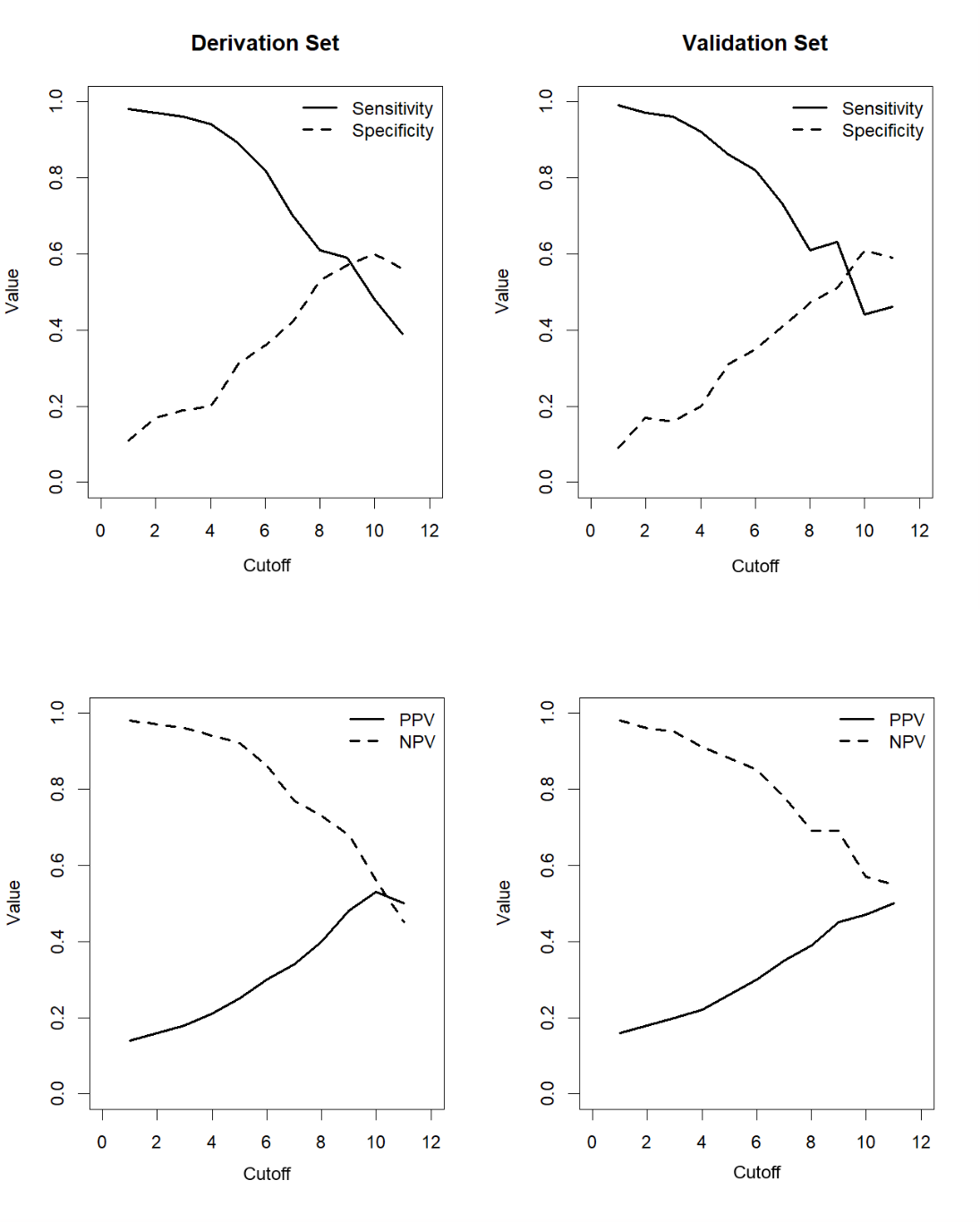


**Figure S3. The sensitivity analysis: comparison between prediction score with additional variables and original models**

The area under the curve of the model with additional variables (0.79, 95% confidence interval; 0.76-0.81) was not significantly different from that of the original Model_1 (0.78; p=0.051).


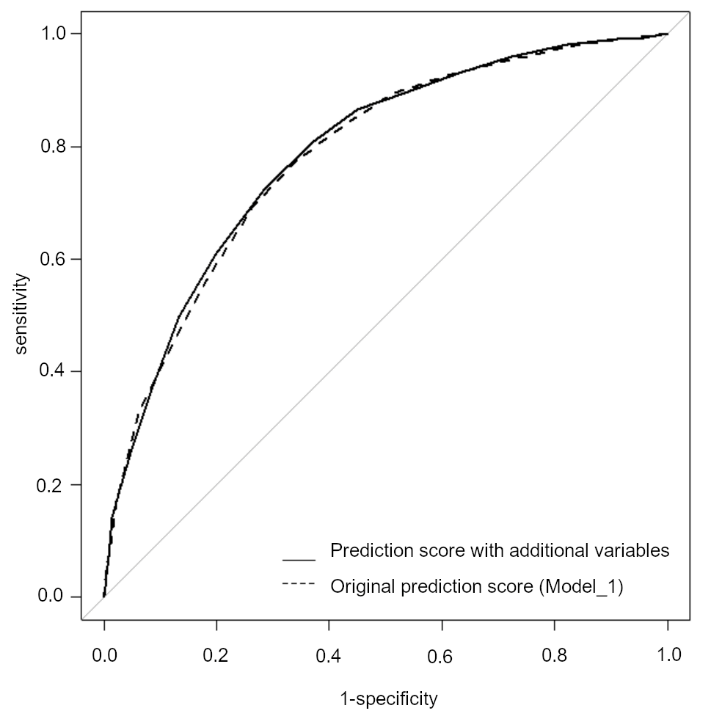


**Supplementary tables**

**Table S1. List of the missing data**

| Variables | Total | Missing |  |
| --- | --- | --- | --- |
| Body temperature (°C) | 7,349 | 1 | 0.01 (%) |
| Heart rate (/min) | 7,349 | 24 | 0.32 (%) |
| Bilirubin (mg/dL) | 7,349 | 1 | 0.01 (%) |
| Lactate (mg/dL) | 7,349 | 91 | 1.24 (%) |
| C-reactive protein (mg/dL) | 7,349 | 37 | 0.50 (%) |

**Table S2. Causative organism of bacteremia and contamination**

|  | Derivation set (n = 3,725) | Validation set (n = 3,471) |
| --- | --- | --- |
| Bacteremia, n (%) | 465 (12) | 503 (14) |
| Gram positive cocci |  |  |
| *Staphylococcus aureus* | 35 (8) | 50 (10) |
| Coagulase-negative Staphylococcus | 9 (2) | 5 (1) |
| *Streptococcus* species | 60 (13) | 64 (13) |
| *Enterococcus* species | 21 (5) | 20 (4) |
| Gram negative rod |  |  |
| *Escherichia coli* | 174 (37) | 178 (35) |
| *Klebsiella* species | 70 (15) | 80 (16) |
| Other Enterobacteriaceae | 30 (6) | 32 (6) |
| *Pseudomonas aeruginosa* | 3 (1) | 12 (2) |
| Anaerobes | 33 (7) | 31 (6) |
| Other organisms | 30 (6) | 31 (6) |
|  |  |  |
| Contamination, n (%) | 40 (1) | 47 (2) |
| Coagulase-negative *Staphylococcus* | 13 (33) | 11 (23) |
| Viridans streptococci | 13 (33) | 12 (26) |
| Anaerobes | 4 (10) | 2 (4) |
| *Enterococcus* species | 2 (5) | 3 (6) |
| *Staphylococcus* *aureus* | 2 (5) | 4 (9) |
| Other streptococci | 2 (5) | 1 (2) |
| *Corynebacterium* species | 1 (3) | 2 (4) |
| *Pseudomonas aeruginosa* | 1 (3) | 0 (0) |
| *Candida* species | 1 (3) | 1 (2) |
| *Bacillus* species | 1 (3) | 0 (0) |
| Unclassified | 1 (3) | 10 (21) |

**Table S3. Comparison of prototype models**

| Model | Variables | AUC | p value |
| --- | --- | --- | --- |
| Model_1 | NLR×4 + Plt×3 + Bil×2 + Lac×2 + Cre×2 + BT×2 + Alb + HR + CRP + WBC | 0.78 | N/A |
| Model_2 | NLR×4 + Plt×3 + Bil×2 + Lac×2 + Cre×2 + BT×2 + Alb + HR +CRP | 0.78 | 0.66 |
| Model_3 | NLR×3 + Plt×2 + Bil×2 + Lac×2 + Cre×2 + BT + Alb + HR | 0.78 | 0.43 |
| Model_4 | NLR×3 + Plt×2 + Bil×2 + Lac×2 + Cre + BT + Alb | 0.78 | 0.15 |
| Model_5 | NLR×2 + Plt + Bil + Lac + Cre + BT | 0.77 | <0.01 |
| Model_6 | NLR×2 + Plt + Bil + Lac + Cre | 0.76 | <0.01 |
| Model_7 | NLR×2 + Plt + Bil + Lac | 0.75 | <0.01 |
| Model_8 | NLR + Plt + Bil | 0.71 | <0.01 |
| Model_9 | NLR + Plt | 0.69 | <0.01 |
| Model_10 | NLR | 0.67 | <0.01 |

Abbreviations: NLR, neutrophil-lymphocyte ratio; Plt, platelet count; Bil, bilirubin; Lac, lactate; Cre, creatinine; BT, body temperature; Alb, albumin; CRP, C-reactive protein; WBC, white blood cell count; AUC, Area under the curve; N/A, not applicable
As our analytical methods, we used multivariate logistic regression to build each model and DeLong’s test to compare their AUC values.

The p values in this table compare the AUC of each model with that of Model 1. Because Model 1 is the reference, no p value is applicable to it.

**Table S4. Prediction performance of each cutoff value**

| Derivation set | | | | | | | |
| --- | --- | --- | --- | --- | --- | --- | --- |
| Cutoff | Sensitivity | Specificity | PPV | NPV | LR+ | LR− |  |
| ≥1 | 0.98 | 0.11 | 0.14 | 0.98 | 1.11 | 0.13 |  |
| ≥2 | 0.95 | 0.27 | 0.16 | 0.98 | 1.30 | 0.17 |  |
| ≥3 | 0.92 | 0.41 | 0.18 | 0.97 | 1.54 | 0.21 |  |
| ≥4 | 0.86 | 0.52 | 0.21 | 0.96 | 1.81 | 0.26 |  |
| ≥5 | 0.77 | 0.67 | 0.25 | 0.95 | 2.34 | 0.35 |  |
| ≥6 | 0.63 | 0.79 | 0.30 | 0.94 | 3.00 | 0.47 |  |
| ≥7 | 0.45 | 0.88 | 0.34 | 0.92 | 3.62 | 0.63 |  |
| ≥8 | 0.27 | 0.94 | 0.40 | 0.90 | 4.69 | 0.77 |  |
| ≥9 | 0.16 | 0.98 | 0.48 | 0.89 | 6.49 | 0.86 |  |
| ≥10 | 0.08 | 0.99 | 0.53 | 0.88 | 7.89 | 0.93 |  |
| ≥11 | 0.03 | 1.00 | 0.50 | 0.88 | 7.01 | 0.97 |  |
| ≥12 | 0.00 | 1.00 | 0.25 | 0.88 | 2.34 | 1.00 |  |
| Validation set | | | | | | | |
| Cutoff | Sensitivity | Specificity | PPV | NPV | LR+ | LR− |  |
| ≥1 | 0.99 | 0.09 | 0.16 | 0.98 | 1.09 | 0.09 |  |
| ≥2 | 0.96 | 0.24 | 0.18 | 0.97 | 1.27 | 0.17 |  |
| ≥3 | 0.92 | 0.37 | 0.20 | 0.97 | 1.46 | 0.21 |  |
| ≥4 | 0.84 | 0.49 | 0.22 | 0.95 | 1.67 | 0.31 |  |
| ≥5 | 0.72 | 0.65 | 0.26 | 0.93 | 2.06 | 0.43 |  |
| ≥6 | 0.59 | 0.77 | 0.30 | 0.92 | 2.58 | 0.53 |  |
| ≥7 | 0.43 | 0.86 | 0.35 | 0.90 | 3.19 | 0.66 |  |
| ≥8 | 0.27 | 0.93 | 0.39 | 0.88 | 3.71 | 0.79 |  |
| ≥9 | 0.17 | 0.96 | 0.45 | 0.87 | 4.77 | 0.86 |  |
| ≥10 | 0.07 | 0.99 | 0.47 | 0.86 | 5.32 | 0.94 |  |
| ≥11 | 0.03 | 0.99 | 0.50 | 0.86 | 5.90 | 0.97 |  |
| ≥12 | 0.00 | 1.00 | 0.40 | 0.86 | 3.93 | 1.00 |  |

Abbreviations: PPV, positive predictive value; NPV, negative predictive value; LR+, positive likelihood ratio; LR−, negative likelihood ratio
